# Challenges of Standard Pediatric Epilepsy Monitoring and the Potential Benefits of Contactless Sensor Technologies: Exploratory Qualitative Study

**DOI:** 10.2196/83009

**Published:** 2026-03-24

**Authors:** Natalie Öhl, Dilbar Strack, Stefan Martin Kusnik, Isabell Link, Marlene Hofmann, Tobias Steigleder, Alina Weisser, Christoph Ostgathe, Regina Trollmann, Maria Heckel

**Affiliations:** 1 Department of Palliative Medicine, University Hospital Erlangen, Friedrich-Alexander-Universität Erlangen-Nürnberg, Werner-von-Siemens Str. 34, Erlangen, 91052, Germany, 49 91127253859; 2 Department of Pediatric and Adolescent Medicine, Division of Pediatric Neurology and Social Pediatrics, University Hospital Erlangen, Friedrich-Alexander-Universität Erlangen-Nürnberg, Erlangen, Germany

**Keywords:** pediatrics, epilepsy, monitoring, technology, qualitative research

## Abstract

**Background:**

Epilepsy is a common neurological condition in children, and accurate detection of seizures and their frequency is essential for diagnosis and treatment. Standard monitoring using electroencephalography alongside clinical observation is often burdensome in pediatric settings, as electrodes can cause discomfort and restrict mobility. Contactless sensor technologies may offer a promising supplement by enabling monitoring without physical contact.

**Objective:**

This study aims to explore challenges in standard pediatric epilepsy monitoring from the perspective of health care professionals and examines the potential benefits and requirements of supplementary contactless sensor technologies in this setting.

**Methods:**

Participant observation of routine processes in standard pediatric epilepsy monitoring was conducted at a German university hospital. Field notes from 40 observed procedures were analyzed using structuring content analysis. Building on these findings, a focus group with pediatric neurologists, nurses, and medical technical assistants (n=6) explored the potential benefits and implementation requirements of contactless sensor technologies. Focus group data were analyzed using focus group illustration maps.

**Results:**

A reference workflow of standard pediatric epilepsy monitoring was derived, revealing psychosocial, medical, and organizational challenges faced by health care professionals. Electroencephalography recordings and clinical observation required considerable reassurance of patients and parents or carers, were vulnerable to movement artifacts and incomplete seizure documentation, and were labor- and resource-intensive. Focus group participants viewed contactless sensor technologies as a potentially valuable supplement by enabling continuous long-term monitoring with minimal additional burden.

**Conclusions:**

By identifying challenges associated with standard pediatric epilepsy monitoring, this study provides a foundation for the needs-based development and implementation of supplementary contactless sensor technologies. Such technologies should be tailored to the clinical setting and designed to address existing burdens, with the potential to complement standard monitoring.

## Introduction

Epilepsy is one of the most common neurological conditions in children and is characterized by an enduring risk of epileptic seizures [1]. Detecting seizures and their frequency is essential for diagnosis, treatment, and therapy monitoring [2]. The current gold standard for epilepsy diagnosis and monitoring is electroencephalography (EEG) alongside clinical observation [3,4].

EEG procedures typically take place in clinical settings and range from short-term recordings of approximately 30 minutes to long-term monitoring over several hours [5]. The electrodes attached to the patient’s head often lead to discomfort and restricted mobility [5,6]. This is particularly relevant in pediatric populations, where resistance to EEG technology may further complicate the procedure. These challenges place considerable demands on health care professionals and add complexity to clinical workflows. While challenges associated with EEG recordings and long-term monitoring in general have been studied [7-9], there remains a lack of empirical knowledge on the specific challenges of standard pediatric epilepsy monitoring in clinical practice from the perspectives of the professionals involved. Qualitative studies on this topic are scarce, and observational studies remain particularly limited [10-12]. Yet, observational approaches are essential in this context, as they allow direct capture of real-world clinical processes, interaction patterns, and workflow constraints, rather than relying on retrospective self-report [13,14].

Various technologies supporting seizure detection have been developed or are under investigation, including video-based approaches, mattress sensors, and mobile applications [6,7]. These systems monitor physiological signals relevant to epilepsy, such as cardiovascular changes, respiration, electrodermal activity, motor activity, audio, eye movements, temperature changes, body pressure, and moisture [6]. Although many are noninvasive, they are typically contact-based [6] and may be difficult to tolerate for children, who cannot meaningfully weigh potential benefits against discomfort and may instinctively try to remove them. Therefore, contactless sensor technologies may be particularly promising in pediatric epilepsy monitoring [6] by reducing discomfort and improving adherence.

One example of a contactless approach is a new type of radar technology based on interferometry [15], currently being developed within federally funded research projects, such as BrainEpP (BMBF: 13GW0295D) and EmpkinS (DFG: CRC 1483‐442419336) [16-18]. Positioned near the child’s chest, for example, under the bed, the radar continuously records the patient’s heartbeat [19-21]. Changes in heart rate and heart rate variability (HRV) are known to occur before and during epileptic seizures [22]. Machine-learning algorithms derive these parameters from radar motion signals [23] and use this information to support seizure detection. In adult populations, the heart rate calculated on the basis of radar data is of high accuracy when compared to the electrocardiogram [16,24,25].

Despite growing interest in contactless seizure monitoring, empirical evidence on how health care professionals perceive their benefits, limitations, and integration into pediatric clinical practice remains limited. Their perspectives are essential to ensure usability, feasibility, and meaningful clinical benefit [6].

To deliver clinical value, innovative technologies such as contactless sensors should be compatible with real-world clinical workflow and constraints [26]. This requires first identifying challenges in current pediatric epilepsy monitoring practices and, building on these insights, understanding how health care professionals envision technological support addressing these challenges. Such a sequential and integrated approach is relevant for the responsible development and implementation of new technologies [27].

Accordingly, this study addresses two research gaps: (1) it explores challenges in standard pediatric epilepsy monitoring at a university hospital epilepsy center from the perspective of health care professionals, to identify opportunities for workflow improvements and technological support; and (2) based on these insights, it examines the potential benefits and requirements of supplementary contactless sensor technologies in this clinical setting from the perspective of health care professionals.

## Methods

### Study Design

**Figure 1.**
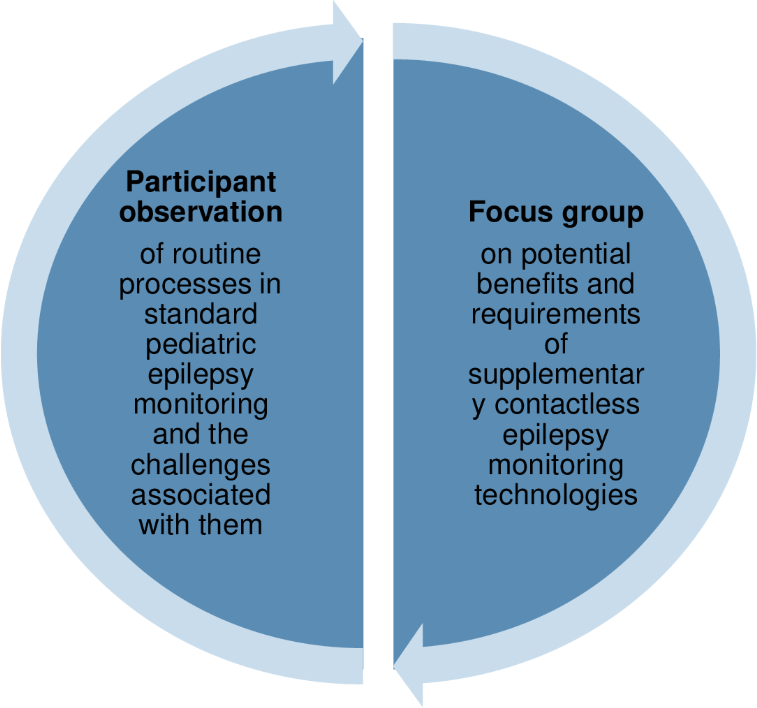
Synthesized methods used in study design.

We used a qualitative design, informed by a constructivist epistemological approach, drawing on principles of ethnography to understand the complex social and organizational processes in pediatric epilepsy monitoring. Between November 2021 and January 2022, we conducted ethnographic fieldwork [13,28] using participant observation [13,14] to examine routine processes in standard pediatric epilepsy monitoring. This method allowed us to capture real-world workflows, interactions, and challenges in context. Building on these observations, we conducted a focus group [29] with health care professionals in June 2022 to explore the potential benefits and requirements of supplementary contactless sensor technologies (Figure 1). The study process adhered to COREQ (Consolidated Criteria for Reporting Qualitative Studies) guidelines (Checklist 1) [30], ensuring transparency and rigor in reporting qualitative research.

### Setting

The data were collected in the pediatric epilepsy center of the Division of Pediatric Neurology at University Hospital Erlangen, Germany. EEG recording (using the 10‐20 system) adhered to the guidelines issued by the DGKN (German Society for Clinical Neurophysiology and Functional Imaging) [31].

### Research Team and Reflexivity

Participant observation and focus group were carried out by IL, a study coordinator, research associate in the Department of Palliative Medicine, and medical technician, in consultation with MaHo, a research associate in the Division of Pediatric Neurology. Prior to conducting the observational study, IL received training in participant observation from an experienced researcher.

### Participant Observation

#### Data Collection

With informed consent, the actions and standard procedures of health care professionals (pediatric neurologists, nurses, and medical technical assistants, MTAs) were observed during routine pediatric epilepsy monitoring. This included diagnostic, long-term, and seizure-detection EEG as well as clinical observation. We chose situations for observation in consultation with the nurse and MTA in charge of the relevant department.

The observer (IL) was not known to the health care professionals beforehand; she introduced herself and gave brief details on the study at the beginning of each situation of interest. The observation included routine EEG recording processes, working environment (structures and organization of the ward; furnishings, equipment, and facilities in patient rooms), type of diagnostic methods, devices, and materials used, and how they were used, roles and responsibilities of the stakeholders involved, communication and collaboration processes, information provision and decision-making, and duration of the monitoring. A guide detailed the components and the focus of the observation (Multimedia Appendix 1). Throughout the observations, health care professionals explained procedures and provided information about processes and experiences. Besides, conversations that arose spontaneously with health care professionals were also documented in the field notes. Between November 2021 and January 2022 (on 8 days, 4 hours each), a total of 40 situations were observed. Documentation was done with field notes and pictures of the technical facilities, with the pictures showing only the equipment and no children or staff. Data collection continued until saturation was reached in terms of varied and typical pediatric epilepsy monitoring situations.

### Data Analysis

On the basis of the field notes, IL created 40 pseudonymized descriptions of the situations and imported them to MAXQDA [32], setting document variables. We used the MAXQDA software (version 20; VERBI Software) for the analysis. Each description comprised details in reference to each component of the observation guide (Multimedia Appendix 1) relating to the care of 1 patient with epilepsy. We ensured interobserver reliability by having multiple researchers independently analyzing the field notes, thereby minimizing potential bias. NO, IL, and MH conducted structured content analysis using content categories as defined by Mayring [33,34]. The units of analysis were determined by the description of situations observed. Coding took place using a subsumption strategy, assigning data to main categories and subcategories. We used the components of the observation guide as deductive main categories and then inductively added further main and subcategories (Table 1).

**Table 1.** Coding scheme for descriptions of situations observed.

Main categories	Subcategories (inductive)
Routine processes (deductive)	Patient admission Medical information and informed consent Patient logistics EEG^a^ electrode placement EEG recording Post EEG procedures EEG interpretation Working routines on the ward
Information sharing (deductive)	Information sharing between parents or carers or patients and pediatric neurologists Information sharing between nurses and pediatric neurologists Documentation
Human-technology interaction (inductive)	Handling movement artifacts and reapplying EEG electrodes Setting markers in EEG recordings
Human-human interaction (inductive)	Instructing and calming patients during EEG electrode placement and recording Instructing and calming parents or carers during clinical observation
Resource requirements (inductive)	Human resources Consumables Time Facilities

a EEG: electroencephalography.

**Figure 2.**
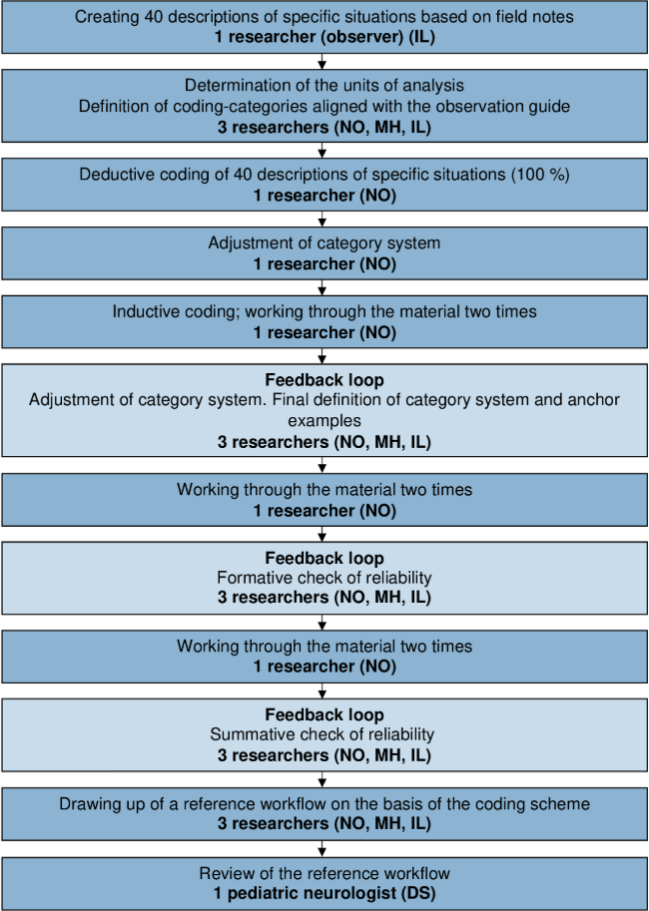
Data analysis using the method of structuring content analysis set out by Mayring [33,34].

Coding was done by one coder (NO) (Figure 2). Due to the heterogeneity of the situations observed, the coder carried out deductive coding of 100 percent of situations before adjusting the system of categories and performing inductive coding. Three feedback loops, involving NO, MH, and IL, took place to enable us to attain the maximum possible intersubjectivity and to verify the categories. The adaptation of the coding scheme was an iterative process that went through the material multiple times. To minimize intersubjective differences in interpretation, we drew up coding guidelines ensuring our codes were clearly defined and included anchor examples. A reference workflow emerged on the basis of the main categories and subcategories; we had a pediatric neurologist (DS) check the workflow for correctness and completeness.

### Focus Group

A focus group discussion with health care professionals was performed to explore the potential integration of supplementary contactless sensor technologies into pediatric epilepsy monitoring, including their perceived benefits and requirements.

### Recruitment of Participants

Health care professionals of the Division of Pediatric Neurology, University Hospital Erlangen, all directly involved in the care of pediatric patients with epilepsy, were invited. Participants included pediatric neurologists (n=4), a nurse (n=1), and an MTA (n=1), thereby covering all relevant perspectives and offering a sample of the broader population of epilepsy care providers. One of the authors (MaHo) approached prospective participants, whom we had specifically identified as meeting these criteria, face to face; IL provided information to those who showed interest in taking part. All those approached gave written consent to participate. The researcher conducting the focus group (IL) was an external research associate who was fleetingly personally known to the participants.

### Data Collection

We created and piloted a focus group guide, informed by preliminary results from the participant observation. The facilitator (IL) received prior training to conduct the focus group. To support the facilitator, MaHo and AW were present during the focus group. The focus group took place in June 2022 at University Hospital Erlangen, with a duration of 60 minutes and was audio-recorded. We printed out key questions in advance of the discussion and made them available to all participants. The focus group commenced with an outline of the research topic and the aims of the research. To support the discussion, we gave a short presentation on an exemplary contactless sensor technology for supporting epilepsy monitoring, involving a flyer detailing the technology. Specifically, we introduced a new type of radar that is currently under development in the research project BrainEpP (BMBF: 13GW0295D).

### Data Analysis

We analyzed the focus group using focus group illustration maps, a method that integrates knowledge mapping principles with focus group procedures [35]. Focus group illustration maps combine data collection and analysis in one unified, structured, and documented process.

### Creating the Maps During the Focus Group

During the focus group, one researcher (AW) documented participants’ statements in real time on a flipchart. Statements were summarized, assigned to thematic clusters, and placed as nodes within a network; connecting lines indicated conceptual links among statements. This graphical documentation supported structuring and condensation of participants’ contributions, preserved relational connections between arguments, and enabled immediate consensual validation, as participants could verify, correct, or expand written content on the spot. By the end of the focus group, a structured and participant-validated visual summary existed for each guiding question.

### Post-Session Refinement

After the focus group, the flipchart graphics were transferred into Miro Board [36], and digital illustration maps were created for each key question. Two researchers (NO and AW) independently reviewed the maps, comparing them with the audio recordings to ensure completeness, correctness, and logical consistency.

As a final step, 2 researchers (NO and IL) aligned the results of the participant observation with the focus group illustration maps to identify connections between the 2 data sources.

### Ethical Considerations

The study was approved by the Ethics Committee of the Faculty of Medicine at Friedrich-Alexander-Universität Erlangen-Nürnberg (350_21 B) on September 20, 2021. The trial was registered with Deutsches Register Klinischer Studien (DRKS; DRKS00027017) on November 16, 2021. All participants received full information on the study and on their right to withdraw consent to participate at any time without giving reasons and were able to ask questions prior to the participant observation and the focus group. All participants gave informed consent to participate in the study. All data were pseudonymized prior to analysis, ensuring that no information could be linked back to individuals, and access to the pseudonymized dataset was restricted to authorized members of the research team only. Participants did not receive any form of compensation for their involvement in the study.

## Results

### Reference Workflow

**Figure 3.**
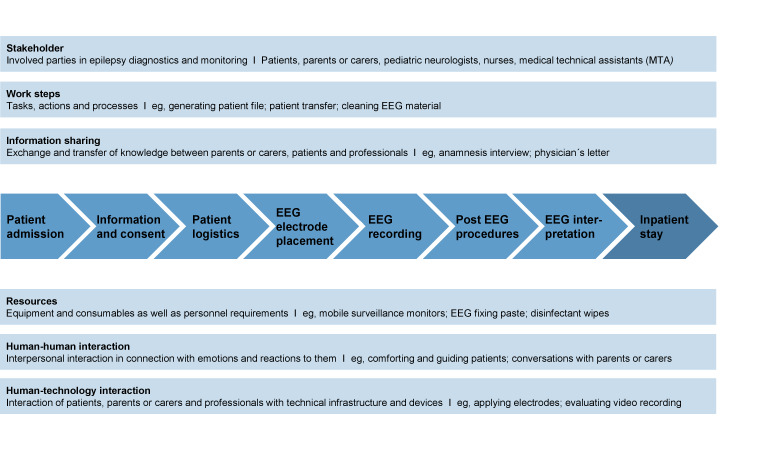
Reference workflow for standard pediatric epilepsy monitoring in a university hospital epilepsy center based on observations of routine processes (own illustration). EEG: electroencephalography.

To contextualize the challenges identified in standard pediatric epilepsy monitoring within a university hospital epilepsy center, we first present a reference workflow that provides an overview of the process. The coding scheme of the participant observation (Table 1) led to this reference workflow (Figure 3). It outlines the clinical stages of pediatric epilepsy monitoring, the stakeholders involved, the work steps, information flows, resources required, and the interactions that characterize routine practice. The process varies depending on the type of EEG (eg, routine vs long-term video EEG procedure) and whether it takes place on an inpatient or outpatient basis. Routine EEGs entail at least 20 minutes of recording, whereas long-term EEGs require approximately 24 hours of recording in the inpatient clinic.

**Figure 4.**
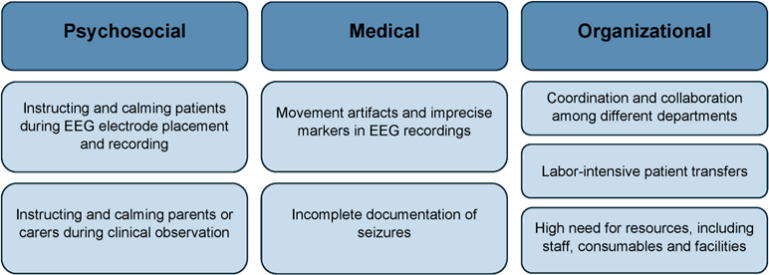
Identified challenges in standard pediatric epilepsy monitoring in a university hospital epilepsy center. EEG: electroencephalography.

Within this reference workflow, psychosocial, medical, and organizational challenges in standard pediatric epilepsy monitoring procedures encountered by health care professionals were identified (Figure 4). These challenges highlight where workflow improvements or supportive technologies could be most beneficial.

### Psychosocial Challenges

#### Instructing and Calming Patients During EEG Electrode Placement and Recording

During the observation, health care professionals explained that patients must remain still during the application of electrodes and the EEG recording, as physical movement can cause artifacts (see “Medical challenges” section below). While many patients tolerated the equipment well, some showed signs of stress or fear, such as screaming, crying, and irregular breathing:

The child starts to wheeze and shows irregular breathing (holding her breath, jerky exhalations). She taps/drums her hands on the examination table cushion.

[S24]

Some resisted the recording technology by shaking their heads or turning away. In several observational situations, MTAs involved in the workflow instructed patients and parents or carers (individuals actively involved in care who have legal parental responsibilities or legal guardianship) to ensure the technology remained in place. Patients with disabilities, limited communication abilities, or cognitive impairments occasionally had difficulties following the MTAs’ instructions. MTAs tried to comfort patients by interacting with them empathetically, using elements of play and physical contact.

Throughout the examination, the medical technical assistant is slightly bent over the child, her arm rests reassuringly on the child’s upper body and she tells a comforting story.

[S22]

MTAs also encouraged parents or carers to calm or distract their children during this process. This was very demanding for MTAs, who had to cope with children crying and screaming while maintaining patience and empathy.

#### Instructing and Calming Parents or Carers During Clinical Observation

In addition to EEG recordings, clinical observation of seizure activity is essential. Health care professionals request parents or carers to document seizures characteristic of the patient’s epilepsy. During the observation, health care professionals noted the generally high motivation of parents or carers to engage in this process. However, they also acknowledged that it represents an additional responsibility for parents or carers in an already stressful situation. Health care professionals explained that parents or carers often experience pressure to do everything “right,” although they are not medically trained, and are acutely aware that their child’s diagnosis may depend on the quality of their observations. Health care professionals explained that they face the challenge of calming parents or carers in this demanding situation while ensuring that the documentation is done correctly. This can be further complicated in cases involving language barriers:

Due to the limited language proficiency of some non-native parents, they do not fully understand their tasks and have difficulties knowing what and how to document.

[S13]

### Medical Challenges

#### Movement Artifacts and Imprecise Markers in EEG Recordings

During the observation, patients’ movements in the course of EEG recordings led to artifacts and thus reduced the recordings’ informational value in medical terms.

The patient is extremely tense, resulting in an EEG that is heavily affected by artifacts.

[S10]

When patients resisted the procedure, electrodes and probes became detached, fell off, and required reapplication; this interrupted the course of monitoring and reduced the overall time it covered. MTAs sometimes struggled to obtain analyzable recordings. They documented movement artifacts that occurred by placing a marker in the recording; the necessity of paying simultaneous attention to several parallel tasks occasionally resulted in markers being set imprecisely.

#### Incomplete Documentation of Seizures

The documentation of epileptic seizures by health care professionals and parents or carers is of crucial significance. However, the observation showed that its completeness was occasionally limited due to the difficulties that even specialists experience in explaining different types of seizures to laypeople. Health care professionals explained that parents or carers lack professional knowledge, which impedes correct documentation. Language barriers were an additional source of misunderstanding. The information loss may impact treatment:

Information on seizures is completely missing in the template and the further diagnosis has to be made without this additional information.

[S13]

Health care professionals explained that specialized epilepsy centers typically video-record long-term EEGs, which enables pediatric neurologists to examine the video material when parents or carers have documented a seizure incompletely. However, it is extremely time-consuming, and not all seizure patterns can be identified.

#### Organizational Challenges

The observation showed that epilepsy monitoring in a pediatric epilepsy center is a labor- and resource-intensive procedure that makes heavy demands on the management of procedures and routines, including appointments and patient transport for EEG recordings.

#### Coordination and Collaboration Among Different Departments

Coordination of appointments for inpatients took place via telephone conversations with the ward. When the ward was busy or an emergency arose, delays could cause inpatients to arrive late. If a patient was late and the monitoring room had been prepared specifically for this patient, it was usually not possible to fill the gap with another patient; the delay caused delays to other patients and occasionally resulted in postponed appointments. This dynamic situation required staff to respond flexibly and make rapid decisions on the order of patients and who to prioritize.

An MTA repeatedly calls the ward and asks whether the patient has already been admitted and explains that all preparations have been made (...). Referring to the digital schedule of appointments, two MTAs begin to discuss which patients’ appointments could be moved forward during the waiting period.

[S15]

#### Labor-Intensive Patient Transfers

The clinical processes vary based on which EEG is required (routine or long-term). Health care professionals explained that, depending on the process used for EEG electrode placement and recording, patients need to be transferred between the pediatric neurology ward, the outpatient clinic, and, on occasion, the EEG monitoring room.

Patient transport depends on the patient’s health status, prior experience, and age, and may require one or two medical assistants, or in special cases with high seizure frequency a licensed nurse.

[S34]

Patient transfers took up a substantial amount of time, reducing flexibility in patient management and contributing to delays.

#### High Need for Resources, Including Staff, Consumables, and Facilities

The process of standard epilepsy monitoring engages a large amount of resources, including staff, consumables, and facilities. The observation showed that the preparation and application of EEGs were particularly labor-intensive, depending on the patient’s ability to cooperate. Consumables needed included abrasive paste and cotton swabs to clean the scalp, conductive or fixing paste for attaching the electrodes, and, in some cases, a syringe to insert conductive paste into the electrode caps; general hygiene material will also be required:

The medical assistant first cleans the patient’s scalp with the abrasive paste and a cotton swab and then attaches the cup electrode to the scalp with the fixing paste*.*

[S16]

Long-term EEGs required specially equipped monitoring rooms. Transfer of patients from the ward to these rooms required mobile surveillance monitors and mobile blood pressure monitors.

#### Potential Benefits of Supplementary Epilepsy Monitoring With Contactless Sensor Technologies

In the participant observation, challenges of standard pediatric epilepsy monitoring from the perspectives of health care professionals were identified. In the subsequent focus group, health care professionals discussed the potential benefits and requirements of using contactless sensor technologies for supplementary monitoring.

Overall, participants discussed several aspects that need to be considered when implementing such technology. They emphasized that training is required not only for clinical staff but also for patients and parents or carers. Without proper instruction, additional workload may arise for clinical staff due to questions from parents or carers. Participants suggested that refresher training should be provided after some time, along with a re-evaluation to gather feedback on needs and any problems encountered. There should also be a clear point of contact for issues related to the use of the technology.

Training and a clear contact person are essential to avoid extra workload and ensure smooth use.

The responsibilities of clinical staff need to be clearly defined. Participants noted that implementing this technology requires additional effort, and clinics must allocate sufficient time and resources to support this new task. Depending on the type of contactless technology and the physiological signals being monitored, participants noted that the constraints and technical limitations must be carefully evaluated and considered during implementation.

#### Continuous Long-Term Epilepsy Monitoring Data

Participants described EEG as a significant, long-established, and irreplaceable method for epilepsy diagnosis. However, contactless sensor technologies were seen as a useful complement to the gold standard of epilepsy diagnosis (EEG and clinical observation), in providing long-term monitoring data. Participants emphasized that such technology can only complement, but not replace, EEG.

Sensors work without requiring direct physical contact with the patient, making monitoring easier and less labor-intensive for staff. Specifically, the radar-based technology introduced might allow monitoring biomarkers such as respiration and HRV. Participants discussed its potential to detect seizures and measure their duration, frequency, and type. They argued that continuous monitoring data over a longer period could support diagnosis and guide treatment more effectively than it does currently.

With continuous monitoring, we could gather information to better understand seizure patterns, manage treatment, and assess how medications are working.

The exemplary introduced radar-based technology, which can detect changes in HRV up to 3 minutes before the onset of the seizure [20]. Participants see a benefit in an alarm that could be programmed to sound when a change in biomarkers takes place, enabling timely intervention. However, they noted that there is a risk of false alarms or frequent alerts even for minor seizures, so the system needs to be carefully calibrated.

There is a risk of constant alarms and continuous beeping, which could cause stress for parents and clinical staff, and place a burden on them.

#### Freedom of Movement for Patients During Monitoring

The potential for contactless sensor technologies to avoid restricting patients’ freedom of movement during monitoring was highlighted. This could help prevent additional emotional distress, especially to pediatric patients, and thus be relieving for the health care professionals involved. Specifically, for the introduced radar technology, participants noted that installing it in the patient’s room or near their bed allows patients to move freely within the radar’s field of view, thereby enhancing patient comfort.

Compared to other monitors, it’s contactless, making it easier to handle with patients causing less physical strain on them.

#### Security for Parents or Carers Observing Seizures

It was suggested that it would be valuable if contactless sensor technologies could automatically document seizures and trigger an alarm. Participants emphasized that parents or carers will continue to play an essential role in pediatric epilepsy diagnosis and monitoring by observing and documenting epileptic seizures; however, such technologies might have the potential to provide them with support and greater certainty in this endeavor. Particularly at night, sensors could reduce anxiety about missing a seizure and reduce the pressure of constant vigilance.

With this technology, we don’t have to rely solely on parents to spot every seizure. It helps make things more objective, reduces anxiety about missing a seizure, and allows parents to sleep more peacefully, even when they are not present.

#### Supplementary Monitoring in the Outpatient Setting

Focus group participants highlighted that contactless sensor technologies could be particularly suitable for monitoring at home. They suggested that long-term epilepsy monitoring data outside the clinical setting could open up new perspectives for epilepsy management. Participants noted that nonintrusive epilepsy monitoring in the child’s home may improve adherence to monitoring and reduce the stigmatization associated with frequent hospital visits. However, acceptance among patients and parents or carers in the outpatient setting was discussed. Participants emphasized that it may depend on the child’s age and on alarm settings, suggesting that alarms might be most useful for longer seizures. They also highlighted several requirements for outpatient use, including reliability, quick and easy operation, clear instructions and training, and the availability of a contact person in case of problems, especially during the night.

A hotline at night for technical problems could be very helpful.

## Discussion

### Principal Findings

Participant observation revealed a broad set of psychosocial, medical, and organizational challenges faced by health care professionals during standard pediatric epilepsy monitoring in a university hospital epilepsy center. Building on these findings, the focus group highlighted how contactless sensor technologies might address selected aspects of these challenges. Overall, such technologies were consistently perceived as a complementary rather than replacement tool for EEG and clinical observation, aligning with literature emphasizing the value of multimodal approaches in epilepsy monitoring [6,7].

### Psychosocial Perspective

Standard pediatric epilepsy monitoring imposes considerable psychosocial demands on health care professionals, who must continuously instruct, reassure, and calm patients and their parents or carers during EEG procedures and clinical observation. This sustained emotional labor contributes to workload and stress in an already demanding clinical environment, with potential implications for care quality and staff burnout [37]. Here, the focus group shows that contactless sensor technologies’ value lies primarily in avoiding additional strain rather than reducing existing burdens.

Observations also highlighted stress among parents or carers due to the fear of missing seizure events, a burden well documented in the literature [10]. Participants suggested that supplementary contactless monitoring could increase families’ sense of security by enabling continuous surveillance, while clearly emphasizing that clinical observation remains essential.

### Medical Perspective

Participant observation identified challenges in standard pediatric EEG procedures, including movement artifacts, imprecise seizure markers, and electrode displacement, particularly in children who have difficulty tolerating electrodes or remaining still. Incomplete seizure documentation further compromised the quality and diagnostic yield, with direct implications for clinical decision-making [38,39]. Focus group participants perceived contactless sensor technologies as potentially valuable for providing continuous long-term data to supplement standard monitoring by capturing additional physiological correlates of seizures and informing clinical decisions. At the same time, such technologies have inherent technical and medical limitations, including susceptibility to motion artifacts, limited seizure-type differentiation [40], and the absence of direct cortical signal acquisition. These constraints vary depending on the underlying physiological signals and algorithms, reinforcing the role of contactless sensors as an adjunct rather than an alternative to EEG. Ensuring technical accuracy, patient comfort, and continuous availability is therefore essential and requires further investigation [7]. While this study primarily explored perceived benefits and requirements related to the noncontact design of such technologies, further research is required to evaluate their medical relevance and performance across different use cases, sensor types, and physiological signals. In line with established clinical EEG practice, the integration of supplementary contactless monitoring technologies requires clearly defined minimum standards to ensure patient safety and data quality [38-40]. These include reliable signal accuracy, continuous availability, clear assistance procedures, predefined contraindications, transparent communication of benefits and risks, and well-defined indications for specific patient groups and seizure types [38-40]. Our findings suggest that addressing these requirements is an important implication to ensure that supplementary contactless technologies meaningfully support clinical care without compromising established neurophysiological standards.

### Organizational Perspective

Standard pediatric epilepsy monitoring was found to be labor- and resource-intensive, requiring high coordination across departments and staff-intensive patient transfers. Participants did not view contactless sensor technologies as a solution to these structural challenges, as standard monitoring procedures would remain necessary. Organizational burdens are therefore more likely to be addressed through workflow or structural changes or through alternative technical or nontechnical innovations [41-44].

### Potential for Outpatient Monitoring

Although this study focused on inpatient monitoring, participants noted the potential relevance of contactless technologies in outpatient settings. This aligns with evidence that long-term home monitoring can complement diagnosis and treatment planning [45], particularly given the variability and underreporting of seizures by patients and parents or carers [46]. While prior studies have explored patient and caregiver preferences for seizure detection devices in outpatient settings [47], pediatric-specific research on home-monitoring burdens and acceptance remains limited. Future research should examine the feasibility, perceived benefits, and user acceptance of contactless technologies among pediatric patients and their families, building on similar work from adult epilepsy care with wearable technologies [48].

### Strengths and Limitations

Research on new digital technologies is challenging and requires methodological innovations [27,49,50]. In this qualitative study, we applied a sequential, integrated design, using participant observation to identify challenges in standard pediatric epilepsy monitoring, followed by a focus group to explore the potential role of supplementary contactless sensor technologies. This ensured that technological innovation is grounded in real-world clinical needs and constraints [26].

A key strength of this study is the use of participant observation conducted by an external researcher, supplemented by verbal explanations from the health care professionals involved, which provides a more objective perspective than relying solely on verbal accounts from those involved in the procedures [13,14]. Observations have proven particularly relevant in the context of health technologies [51], as they allow researchers to move beyond subjective intended use and capture actual practices. However, the observer’s presence and concurrent explanations may have influenced behavior.

Subjective bias was mitigated through a structured observation guide and by involving an external researcher rather than a member of the pediatric neurology department. Additionally, the observer’s background in medical engineering may have shaped attention toward technical aspects, which we tried to balance by following the observation guide and consulting with a neuropediatrician when needed.

The focus group captured expert perspectives from health care professionals directly involved in pediatric epilepsy care, ensuring high clinical relevance. However, the exclusively professional perspective and the predominance of pediatric neurologists may have influenced the findings. Future studies should therefore include a broader range of health care professionals, as well as patients and parents or carers, to capture diverse experiences and expectations.

This study was conducted at a single tertiary hospital in Germany, where resources, staffing, and EEG logistics may differ from other settings. Potential differences in such contextual factors and internal processes make it impossible to generalize the findings completely or apply them directly to other epilepsy centers; however, we may safely assume that the challenges identified can reasonably be expected in comparable settings. Future research should include multisite studies across different care contexts (eg, outpatient or non-university hospital settings) to capture a broader range of contextual factors.

### Conclusions

We have found standard pediatric epilepsy monitoring in a university hospital epilepsy center to entail psychosocial, medical, and organizational challenges for health care professionals. Having identified these, we consider that we have created an important foundation for the development of new supportive contactless sensor technologies for this setting. Any such new technology needs to fit into the specific care setting and seek to address the existing challenges and burdens in order to deliver a needs-based benefit. The capacity of contactless sensor technologies to monitor seizures without physical contact with the patient suggests that they have great potential to support standard pediatric epilepsy monitoring in both in- and outpatient settings with long-term monitoring data.

## Supplementary material

10.2196/83009 Multimedia Appendix 1 Observation guide.

10.2196/83009 Checklist 1 COREQ Checklist.

## Abbreviations

COREQ: Consolidated Criteria for Reporting Qualitative Studies
EEG: electroencephalography
HRV: heart rate variability
MTA: medical technical assistant
