# Supplementary material for: Challenges of Standard Pediatric Epilepsy Monitoring and the Potential Benefits of Contactless Sensor Technologies: Exploratory Qualitative Study
Source: J Med Internet Res. 2026 Mar 24;28:e83009. doi: 10.2196/83009 (PMC13012698; doi:10.2196/83009)
Supplement: Multimedia Appendix 1 [file jmir-v28-e83009-s001.docx]

| Observation guide | |
| --- | --- |
| Participant ID / pseudonym |  |
| Place of observation |  |
| Day of observation |  |
| Observation period |  |
| Observation and documentation by |  |
| Protocol created on |  |

**Situation no. X**

Routine EEG recordings processes and working environment

- Structures and organization of the ward
- Furnishings
- Equipment and facilities in patient rooms
- Type of diagnostic methods
- Devices, and materials used
- Roles and responsibilities of the stakeholders involved
- Communication and collaboration processes
- Information provision and decision-making and duration of the monitoring
